# Drug Treatment in Older People before and after the Transition to a Multi-Dose Drug Dispensing System–A Longitudinal Analysis

**DOI:** 10.1371/journal.pone.0067088

**Published:** 2013-06-24

**Authors:** Susanna M. Wallerstedt, Johan Fastbom, Kristina Johnell, Christina Sjöberg, Sten Landahl, Anders Sundström

**Affiliations:** 1 Department of Clinical Pharmacology, Sahlgrenska University Hospital, Göteborg, Sweden; 2 Aging Research Center, Karolinska Institutet and Stockholm University, Stockholm, Sweden; 3 Department of Geriatrics, Sahlgrenska University Hospital/Mölndal, Mölndal, Sweden; 4 Department of Geriatrics, Sahlgrenska academy, Mölndal, Sweden; 5 Centre for Pharmacoepidemiology, Karolinska Institutet, Stockholm, Sweden; Universidade Federal do Rio de Janeiro, Brazil

## Abstract

**Background:**

An association has been found between multi-dose drug dispensing (MDD) and use of many drugs. The aim of this study was to investigate the nature of this association, by performing a longitudinal analysis of the drug treatment before and after the transition to MDD.

**Methods:**

Inclusion critera in this register-based study were inhabitants in Region Västra Götaland, Sweden, who, at ≥65 years of age and between 1^st^ July 2006 and 30^th^ June 2010, filled their first MDD prescription. For each individual, prescribed drugs were estimated at three month intervals before and after (maximum 3 years, respectively) the first date of filling an MDD prescription (index date).

**Results:**

A total of 30,922 individuals matched the inclusion criteria (mean age: 83.2 years; 59.9% female). There was a temporal association between the transition to MDD and an increased number of drugs: 5.4±3.9 and 7.5±3.8 unique drugs three months before and after the index date, respectively, as well as worse outcomes on several indicators of prescribing quality. When either data before or after the index date were used, a multi-level regression analysis predicted the number of drugs at the index date at 5.76 (95% confidence limits: 5.71; 5.80) and 7.15 (7.10; 7.19), respectively, for an average female individual (83.2 years, 10.8 unique diagnoses, 2.4 healthcare contacts/three months). The predicted change in the number of drugs, from three months before the index date to the index date, was greater when data before this date was used as compared with data after this date: 0.12 (0.09; 0.14) versus 0.02 (−0.01; 0.05).

**Conclusions:**

After the patients entered the MDD system, they had an increased number of drugs, more often potentially harmful drug treatment, and fewer changes in drug treatment. These findings support a causal relationship between such a system and safety concerns as regards prescribing practices.

## Introduction

Although dose dispensing systems are widespread over the world [1], scientific evidence is scarce [2], [3]. Indeed, beneficial effects have not been proven [3], illustrated, for example, by the inconsistent results for effects on compliance [4], [5]. On the contrary, recent research has indicated safety concerns regarding the prescribing of drugs to patients within such systems. For example, the odds for potentially harmful drug treatment according to polypharmacy indicators were 3.58 (≥10 drugs) to 5.48 (≥3 psychotropics) times greater for patients aged ≥65 years within the Swedish multi-dose drug dispensing (MDD) system, after adjustments for age, sex, burden of disease, and residence [6]. Furthermore, drug orders were more seldom changed within this MDD system [7], a finding which indicates that such systems may diminish physicians’ reconsideration of drug treatment. In addition, medication errors have been reported to be almost six times as common in older patients with MDD [8].

If there is a causal relationship between MDD and potentially harmful drug treatment, this is alarming since the prescriber rather than the nursing and pharmacy services accounts for the majority of severe medication errors [9]. However, no conclusions on causality can be drawn since previous controlled studies, to the best of our knowledge, have applied a cross-sectional or a case-control design. Although a randomized controlled design may be preferable when to evaluate causality, this kind of design may not be feasible when it comes to the effects of MDD on prescribed drugs. Indeed, an MDD system may be already implemented. In Sweden, for example, about eleven per cent of people aged ≥65 years use such a system (described elsewhere) [7], because they have difficulties in handling their drugs due to impaired physical or cognitive function.

A strategy to further investigate the association between MDD and drug treatment may be to analyze drug treatment over time in register data, i.e. a longitudinal analysis on the individual level encompassing time before and time after the transition to MDD. Indeed, such a strategy may be an alternative to a randomized controlled design when the latter is not feasible. Thus, the aim of the present study was to analyze drug treatment over time in individuals, aged ≥65 years, before and after they entered an MDD system.

## Materials and Methods

### Ethics Statement

All data in the registers at question are recorded without written consent from the patients. Before data were extracted for the purpose of this study, approval was obtained from the Regional Ethical Review Board in Gothenburg, which waived informed consent (Dnr: 782-11).

### Data Extraction

Data for the present study were extracted from three individual-based registers, and linked by the unique personal identity number: the Swedish Population Register at the Statistics Sweden, the Swedish Prescribed Drug Register at the National Board of Health and Welfare, and Vega (a regional register including healthcare consumption and diagnoses according to the International Statistical Classification of Diseases and Related Health Problems, ICD-10). The study comprised data from 1^st^ July 2005 to 31^st^ December 2010 (study period).

### Study Population

The study population was extracted from individuals residing in the Region Västra Götaland at any time during the study period. All individuals who entered the MDD system between 1^st^ July 2006 and 30^th^ June 2010 at the age of ≥65 years were included. Thus, at least one year with drugs prescribed by ordinary prescriptions was ascertained (1^st^ July 2005 to 30^th^ June 2006), as well as a follow-up of at least six months after the transition to MDD. Since data on burden of disease was obtained from a regional register, individuals who moved into/out of this region during the study period were excluded. Individuals whose personal identity number had been used before were also excluded to avoid mismatching between registers.

### Drug Treatment

**Figure 1:**
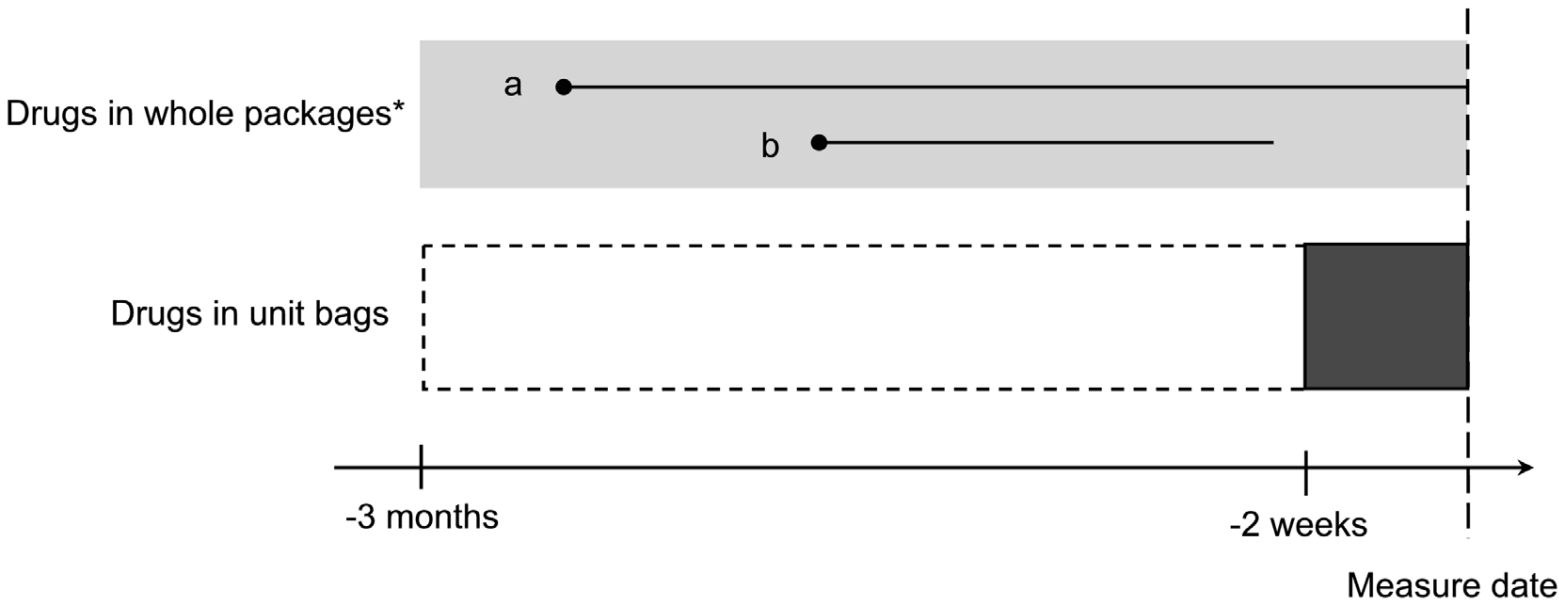
Illustration of the method used to estimate the medication list. A medication list was estimated at the transition to multi-dose drug dispensing (MDD) as well as at up to 12 measure dates before and after this date, respectively, with three month intervals. For drugs purchased in whole packages (prescribed via ordinary prescriptions or MDD) at any time during the three month period preceding the measure date (light grey bar), the duration of treatment with the drug was estimated according to (i) the date of filling the prescription, (ii) the amount of drug dispensed, and (iii) the prescribed daily dose, or, if not available, the mean daily dose in the study population. If the duration of the drug covered the measure date, the drug was included in the medication list, i.e. drug a, but not drug b. Concerning drugs prescribed via the MDD system and dispensed into units bags, all drugs purchased within the time frame of the dark grey bar, but not the dotted one, were included in the medication list. *All drugs prescribed vid ordinary prescription and about 50% of drugs prescribed via the MDD system [12] are delivered in whole packages.

All estimations of drug treatment were performed according to an established method [10] that is also employed by the National Board of Health and Welfare. In short, the method implies that a medication list is constructed for each measure date (Figure 1). For each individual, the first date of filling an MDD prescription was defined as the index date. Drug treatment was evaluated at three month intervals before and after this date, up to 36 months, respectively. After the index date, individuals were censored, if applicable, when they returned to ordinary prescriptions and at death.

Before the index date, the patients received their drugs via ordinary prescriptions. At each measure date, a medication list was constructed according to the filled prescriptions registered in the Swedish Prescribed Drug Register during the three month period preceding the date in question. The register was initiated on 1^st^ July 2005 and, thus, the earliest measure date was 30^th^ September 2005. The rationale for the three month time frame was Swedish regulations, which allow drug use for a maximum of three months to be reimbursed at one purchase occasion. To assess if a drug was to be included in the medication list, i.e. if the purchase covered treatment at a specific measure date, we used (i) the date of filling the prescription, (ii) the amount of drug dispensed, and (iii) the prescribed dosage. When prescribed dosage was incomplete or missing, the mean daily dose for the specific drug in the study population was used. For drugs prescribed as needed we assumed a dosage of 50% of that for regular drugs. Moreover, we assumed a daily dose of 1 defined daily dose (DDD) [11] for drugs for external use and for the eye. Drugs were aggregated at the substance level of the Anatomical Therapeutic Chemical (ATC) classification system (7 digits) [11].

Within the MDD system, prescribed drugs are either dispensed into unit bags with prescriptions filled every fortnight, or delivered in original packages. Therefore, from the index date and onwards, we included dose-dispensed drugs in the medication list if filled within 14 days before the measure date in question. For drugs delivered in whole packages (about 50% of drugs prescribed vid the MDD system) [12], we included drugs in the medication list according to the method described above for ordinary prescriptions. As the Swedish Prescribed Drug Register does not include prescribed dosages for patients with MDD, we assumed the prescribed dosage to be the mean daily dose in the population.

To further explore the changes in prescribed drugs that occur at the transition to MDD, the medication list of each measure date was evaluated according to indicators of prescribing quality used by the Swedish National Board of Health and Welfare, that is, use of *Ten or more drugs*, *Three or more psychotropics*, *Long-acting benzodiazepines*, *Drugs with anticholinergic effects*, *Drug combinations that should be avoided* [13], and *Antipsychotics* (Table 1). Moreover, a descriptive analysis of the medication list was applied to the subgroup of patients still filling MDD prescriptions three months after the index date. In this analysis, we identified the drugs which were most frequently added to the medication list at the index date.

**Table 1:** Description of the indicators of prescribing quality.

Indicator	Included drugs	ATC-code^1^
Ten or more drugs	All drugs	
Three or more psychotropics	Antipsychotics	N05A
	Anxiolytics	N05B
	Hypnotics and sedatives	N05C
	Antidepressants	N06A
Long-acting benzodiazepines	Diazepam	N05BA01
	Nitrazepam	N05CD02
	Flunitrazepam	N05CD03
Drugs with anticholinergic effects	(Anticholinergic) drugs for functional gastrointestinal disorders	A03AB, A03BA, A03BB
	(Anticholinergic) antiemetics	A04AD
	Antiarrythmics class Ia	C01BA
	Urinary antispasmodics	G04BD
	Opioids in combination with antispasmodics	N02AG
	Anticholinergic (anti-Parkinson drugs)	N04A
	Low potency antipsychotics	N05AA, N05AB04, N05AF03
	Hydroxyzine	N05BB01
	Non-selective monoamine reuptake inhibitors (antidepressants)	N06AA
	Antihistamins	R05CA10, R06AA02, R06AB, R06AD, R06AX02
Drug combinations that should be avoided	D-interactions according to the Swedish Physicians’ Desk Reference [13]	
Antipsychotics	Antipsychotics	N05A

1 ATC-code, Anatomical Therapeutical Chemical classification code [11].

### Burden of Disease

In order to investigate the longitudinal health course of a patient, the number of unique diagnoses (ICD-10-diagnoses, 3 digits) in hospital and primary care were summarized cumulatively for each individual at three month intervals. Only codes starting with A to T were included. Hence, we excluded codes starting with V to Y (external causes of morbidity and mortality), Z (factors influencing health status and contact with health services), or U (codes assigned for special purposes). At the first measure date, unique diagnoses were extracted from the year preceding this date. For the following measure dates, additional unique diagnoses were counted and added. In addition, we identified the hospital diagnoses which most frequently preceded the transition to the MDD system. The longitudinal health course was also investigated by extracting the number of healthcare contacts during which prescribing changes primarily occur, that is, hospital admissions as well as physician contacts in outpatient care.

### Statistical Analysis

The data were handled using SAS Enterprise Guide 4.3 (SAS Institute Inc, Cary, NC, USA), and the statistical analyses were performed using SPSS 17.0 (SPSS, Chicago, IL, USA). A descriptive longitudinal analysis was performed regarding the drug treatment, the number of unique ICD-10 diagnoses, and the number of healthcare contacts. We also analyzed the proportion of the patients at each measure date who had the same number of drugs at that specific date compared with the previous one. Multi-level regression models were constructed to predict the total number of drugs at the index date as well as the change in the number of drugs between the measure date preceding the index date and the index date. Each measure date represented level 1 and individuals were level 2. The parameter estimates were based on data before or after the transition to MDD, respectively. Hence, in order to predict results at the index date, a prediction forwards was made for the data before the index date, and a prediction backwards for the data after this date. In order to allow the figures obtained at this date to represent the results for an average individual, that is, an individual of mean age with the mean number of unique ICD-10 diagnoses and the mean number of healthcare contacts during the three month periods between the measure dates, grand-mean centered values were calculated and used in the models. The intercept was estimated with time in a random effects model. The other covariates were included as fixed main effects only. The maximum likelihood estimation procedure was used to estimate the parameters of the models. To test whether addition of another covariate improved the model, the difference in -2log likelihood values was tested for chi-squared distribution.

## Results

**Figure 2:**
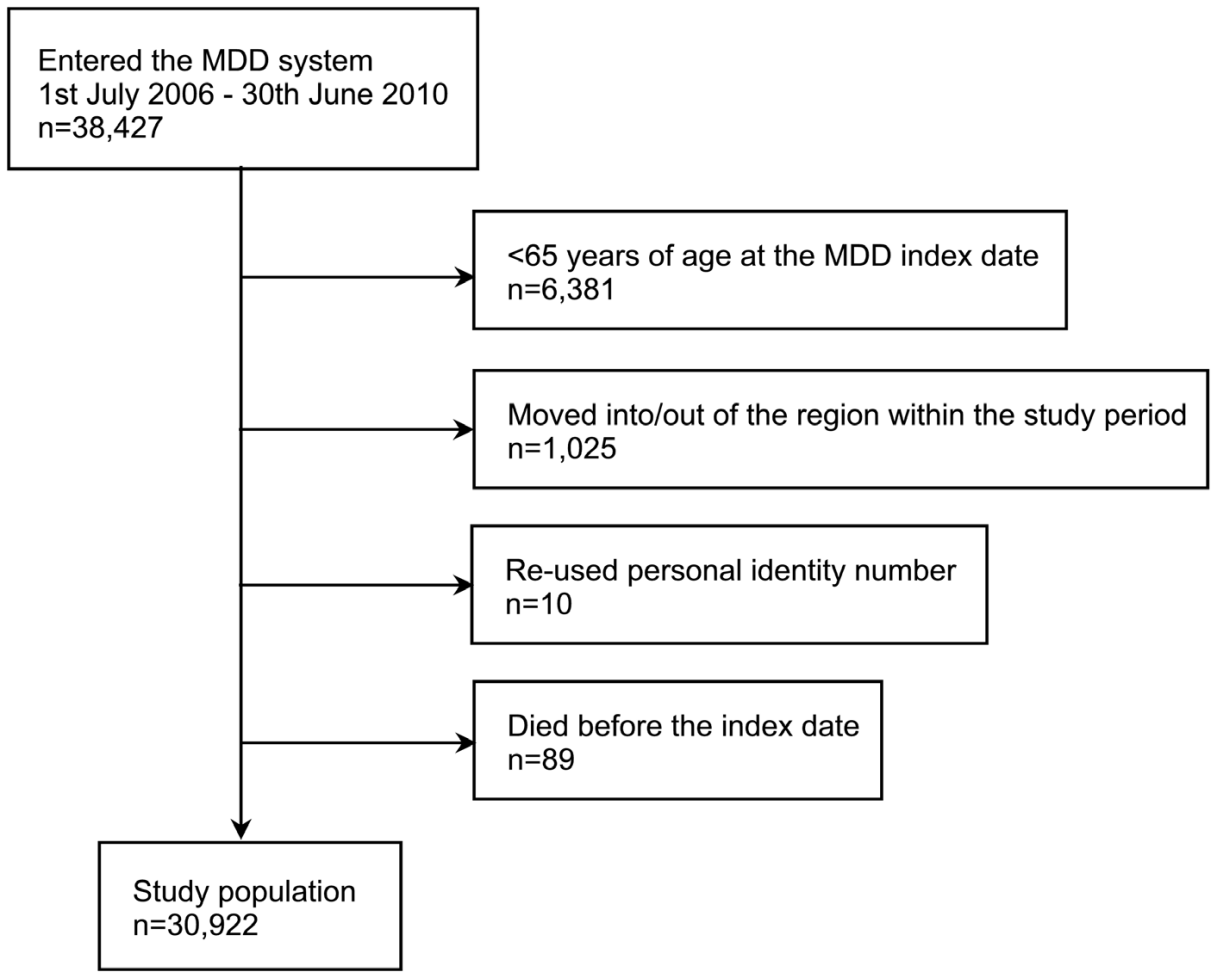
Flowchart of the study population. The study population was extracted from individuals residing in the Region Västra Götaland at any time during the study period (1^st^ July 2005–31^st^ December 2010).

A flowchart of the study population is presented in Figure 2. A total of 30,922 individuals were included in the analyses (mean age±standard deviation (SD): 83.2±7.2 years; 59.9% female).

**Figure 3:**
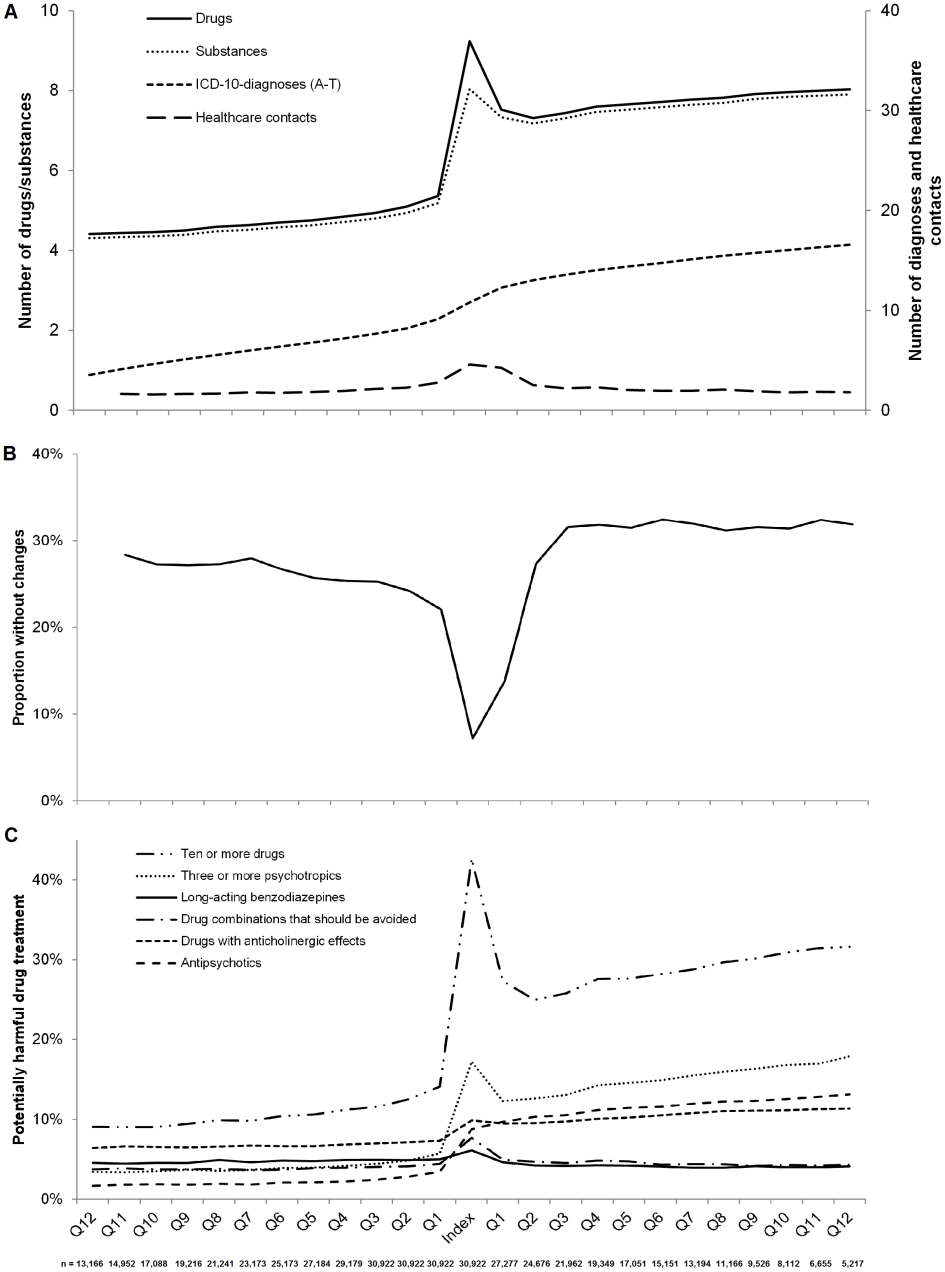
Longitudinal results for 30,922 individuals at three month intervals before and after the transition to multi-dose drug dispensing (index date). (A) The mean number of unique drugs and substances (primary y-axis), the cumulative number of unique ICD-10-diagnoses, and the number of healthcare contacts within each three month period (secondary y-axis). (B) The proportion of the patients without a change in the number of drugs at a specific measure date compared with the previous one. (C) The proportion of the patients who had potentially harmful drug treatment according to indicators of prescribing quality. The individuals were censored when they returned to ordinary prescriptions and after death. The number of individuals included at each specific measure date is presented below the figures.

In all, drug treatment was estimated at 493,396 occasions, at a maximum of 25 measure dates with three month intervals for each individual. The patients were treated with up to 40 unique drugs concomitantly. The longitudinal course of the number of unique drugs and unique substances is presented in Figure 3A, along with the cumulative number of unique ICD-10 diagnoses and the number of healthcare contacts within each three month period. A peak in the number of drugs was observed at the index date: 9.2±4.8 drugs and 8.0±4.0 substances, respectively. Three months before and three months after the index date, the number of unique drugs was 5.4±3.9 and 7.5±3.8, respectively. The increase in the number of drugs was maintained during the follow-up. The proportion of the individuals who, at three month intervals, had the same number of drugs at a specific measure date compared with the preceding one, is presented in Figure 3B.

In Figure 3C, the longitudinal results on indicators of prescribing quality are presented. A peak in the proportion of individuals with potentially harmful drug treatment at the index date as well as a maintained elevated level thereafter was observed for *Ten or more drugs* and *Three or more psychotropics*. A peak at the index date and a subsequent dip to previous level or lower was observed for *Longacting benzodiazepines* and *Drug combinations that should be avoided*. An increase at the index date without a subsequent decline was observed for *Drugs with anticholinergic effects* and *Antipsychotics*.

**Figure 4:**
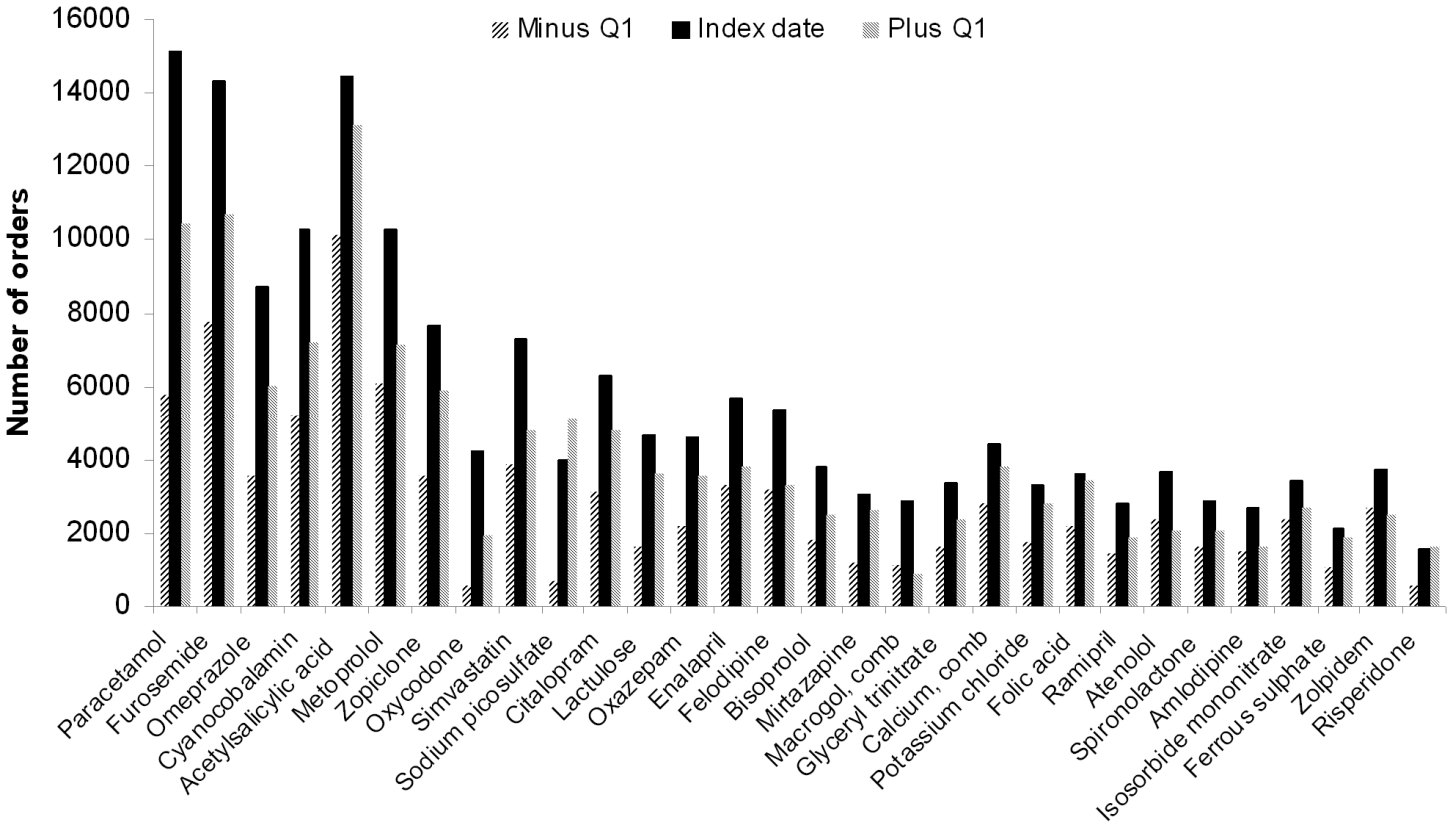
ATC substances that increased by more than 1000 orders at the index date in individuals alive three months after the index date still filling multi-dose drug dispensed prescriptions.

In all, 9,990 patients (32.3%) had received inpatient care within 14 days before the index date. The most frequently registered ICD-10 diagnoses on these occasions were cerebral infarction, hip fracture, heart failure, and myocardial infarction. A total of 8.9% of all individuals had either of these diagnoses. Paracetamol, furosemide, cyanocobalamin, omeprazole, and simvastatin were the drugs most frequently added to the medication list when an individual entered the MDD system (Figure 4).

Five multi-level regression models were constructed; each with an additional covariate included (Table 2). Regarding the number of drugs, but not the change in the number of drugs, the models were significantly improved for each parameter that was added. For an average female individual (83.2 years of age with 10.8 unique ICD-10 diagnoses and 2.4 healthcare contacts within three month periods), the model predicted the number of drugs at the index date, based on either data before or data after this date, at 5.76 (95% confidence limits: 5.71; 5.80) and 7.15 (7.10; 7.19), respectively. This model also revealed that the predicted change in the number of drugs, from three months before the index date to the index date, was greater when data before this date was used as compared with data after this date: 0.12 (0.09; 0.14) versus 0.02 (−0.01; 0.05).

**Table 2:** The predicted number of drugs at the index date and the predicted change in the number of drugs at the index date compared with the preceding measure date.

	Variables included in the model	Number of drugs			Change in number of drugs	
		Before	After		Before	After
Model 1	Time	5.28 (5.25; 5.32)	7.31 (7.27; 7.35)		0.20 (0.17; 0.22)	0.02 (−0.003; 0.05)
Model 2	TimeDiagnoses	5.59 (5.56; 5.62)	7.13 (7.09; 7.17)		0.21 (0.18; 0.23)	0.02 (−0.002; 0.05)
Model 3	TimeDiagnosesHealthcare contacts	5.57 (5.54; 5.60)	7.05 (7.01; 7.09)		0.12 (0.10; 0.15)	0.02 (−0.004; 0.05)
Model 4	TimeDiagnosesHealthcare contactsAge	5.57 (5.34; 5.60)	7.05 (7.01; 7.09)		0.12 (0.10; 0.15)	0.02 (−0.002; 0.05)
Model 5	TimeDiagnosesHealthcare contactsAgeFemale sex	5.76 (5.71; 5.80)	7.15 (7.10; 7.19)		0.12 (0.09; 0.14)	0.02 (−0.01; 0.05)

The parameter estimates are based on either data before or data after the index date. Values, given with 95% confidence limits within parentheses, represent parameter estimates at the index date for an average individual, who had 10.8 unique ICD-10 diagnoses, 2.4 healthcare contacts during the three month period between the measure dates, and a mean age of 83.2 years.

## Discussion

In this longitudinal study, we show a temporal association between the transition to MDD and an increased number of drugs in the medication list. Indeed, the patients received about two more drugs after the transition, and this increase was maintained throughout the follow-up. We also show that the patients more often had potentially harmful drug treatment after they entered the system. Multi-level regression analyses, adjusted for burden of disease, age, and sex, confirm that the transition to MDD is associated with an increased number of drugs. The initial addition of drugs at the index date may be the most prominent underlying factor for these results. Further, the temporal association between the transition to MDD and an increased proportion of patients with the same number of drugs at consecutive measure dates indicates that the drug treatment may be more seldom reconsidered within such a system. Indeed, the predicted change at the index date (the number of drugs at the index date minus the number of drugs three months before this date) was smaller when data after the transition was used for the estimation.

The peak observed at the index date may, at least partly, be explained by the definition of this date, which represents the date of the first filled MDD prescription. Since such prescribing is initiated by a physician, a clinical event that requires drug treatment may precede the physician contact. Indeed, there was a slight temporal increase in the slope of the curve regarding number of unique diagnoses around the index date, probably drawn by the increased number of healthcare contacts during this period. In addition, the most common events preceding the index date were diagnoses which may all may lead to the initiation of drug treatment, for example cerebral infarction, hip fracture, heart failure, and myocardial infarction. Indeed, cardiovascular drugs and analgesics were among the drugs most frequently added to the medication list when the patients entered the MDD system.

Our longitudinal findings support a causal relationship between MDD and an extended medication list. Thus, we have further characterized the association between MDD and polypharmacy, which has been reported previously [6]. Furthermore, our results imply causality between MDD and potentially harmful drug treatment according to indicators of prescribing quality, where non-characterized associations have been reported previously [6], [14], [15], [16]. This was most prominent for the indicators *Ten or more drugs*, *Three or more psychotropics*, *Drugs with anticholinergic effects* and *Antipsychotics*. In particular, the results on the quality indicator *Antipsychotics* may call for attention, as well as the finding that risperidone was one of the drugs most frequently added to the medication list at the transition to MDD; these drugs have been associated with an increased risk of death in people with dementia [17].

A previous study reported that the odds for a drug to stay unchanged during a six month period were greater if prescribed via the MDD system than via ordinary prescriptions [7]. This measure may be seen as a surrogate variable for drug treatment reconsideration. Our results indicate a causal relationship between MDD and fewer changes in drug treatment; after adjustments for relevant covariates, the predicted change in the number of drugs at the index date, compared with the preceding measure date, was significantly smaller when data after the transition was used for the estimations.

Underlying mechanisms for our findings can only be speculated upon. Some previous studies indicate that an MDD system may reduce medication errors [18], [19] and provide a better overview of a patient’s medication list [20], [21]. Thus, it cannot be excluded that the medication lists in the present study may be appropriate at the individual level, although extended and more often potentially harmful according to indicators. Other studies, on the other hand, indicate the opposite, namely that medication errors are as common or even more common for patients with MDD [8], [22], [23], [24]. Indeed, MDD was the main risk factors for medication errors at transitions in healthcare [25]. Furthermore, the experienced increased workload for general practitioners for prescribing within such systems [20], [21] may affect prescribing practices and reduce reconsideration of drug treatment.

A strength of the present study is that is comprises all individuals in our region who, at ≥65 years of age and during a four year period, filled their first MDD prescription. The large number of individuals, as well as the fact that few individuals were excluded, makes it reasonable to generalize from the results. In addition, our longitudinal approach to analyze drug register data linked to other individual-based register data may represent a valuable tool to further investigate causality hypotheses arisen from cross-sectional and case-control studies, when randomized controlled studies are not feasible. Indeed, our approach implies that each individual is compared with his/herself, before and after the transition, and the comparisons are thus made within a population, and not between different populations. Hence, the risk of confounding by indication is reduced. Moreover, in the multi-level regression analyses, we have included covariates that are important for drug treatment, that is, age, sex, and burden of disease, measured as the cumulative number of unique diagnoses and the number of healthcare contacts.

Limitations of this study include confounding by indication. Although we have tried to minimize this problem, we cannot exclude that the differences observed in the drug treatment at the transition to MDD may be due to factors that have not been considered in the analyses. Indeed, a new healthcare structure, forcing every individual to choose a specific primary care provider, was introduced in Sweden on 1^st^ January 2010. Such healthcare reforms may affect longitudinal results. However, the pattern of an increased number of drugs at the transition to MDD was similar when the results were analyzed according to year of transition (data not shown). Further, we evaluated drug treatment in terms of the number of drugs in the medication list and the outcomes on indicators of prescribing quality. Thus, evidently, an added drug may cancel out a withdrawn drug. Moreover, although the indicators are established outcomes frequently used to describe prescribing practices, the association with quality of drug treatment at the individual level has not been established [26]. Thus, we do not know if the number of drugs or the indicators of prescribing quality can differentiate between appropriate and inappropriate drug treatment. Another limitation is that the method for drug treatment estimations involves uncertainties. For example, medications prescribed as needed may be consumed to a greater or a smaller extent than 50% of the DDD, and may thus not be captured correctly, e.g. epinephrine for treatment of anaphylaxis.

## Conclusion

This study indicates a causal relationship between the transition to MDD and an increased number of drugs, potentially harmful drug treatment, and a reduced reconsideration of drug treatment. Thus, such a system may imply safety concerns regarding the prescribing of drugs. The results should be of interest for health care decision makers and prescribers in countries that already have, or plan to introduce, dose dispensing systems and to people who design the prescribing properties within these systems.
